# Cep44 functions in centrosome cohesion by stabilizing rootletin

**DOI:** 10.1242/jcs.239616

**Published:** 2020-02-21

**Authors:** Delowar Hossain, Sunny Y.-P. Shih, Xintong Xiao, Julia White, William Y. Tsang

**Affiliations:** 1 Institut de Recherches Cliniques de Montréal, 110 avenue des Pins Ouest, Montréal, Québec H2W 1R7, Canada; 2 Division of Experimental Medicine, McGill University, Montréal, Québec H3A 1A3, Canada; 3 Faculté de Médecine, Département de pathologie et Biologie Cellulaire, Université de Montréal, Montréal, Québec H3C 3J7, Canada

**Keywords:** Centrosome, Cohesion, Splitting, Cep44, Rootletin

## Abstract

The centrosome linker serves to hold the duplicated centrosomes together until they separate in late G2/early mitosis. Precisely how the linker is assembled remains an open question. In this study, we identify Cep44 as a novel component of the linker in human cells. Cep44 localizes to the proximal end of centrioles, including mother and daughter centrioles, and its ablation leads to loss of centrosome cohesion. Cep44 does not impinge on the stability of C-Nap1 (also known as CEP250), LRRC45 or Cep215 (also known as CDK5RAP2), and vice versa, and these proteins are independently recruited to the centrosome. Rather, Cep44 associates with rootletin and regulates its stability and localization to the centrosome. Our findings reveal a role of the previously uncharacterized protein Cep44 for centrosome cohesion and linker assembly.

## Abstract (precis)

**Highlighted Article:** Maintenance of centrosome cohesion is critical for the fidelity of cell division. Here, we show that the novel protein Cep44 plays a unique role in the cohesion process through stabilizing rootletin.

## INTRODUCTION

The centrosome is the major microtubule-organizing center in higher eukaryotes (Bornens, 2012). It is composed of two centrioles, the mother and daughter centrioles, surrounded by the pericentriolar material (PCM), which nucleates and extends microtubules. During G1 phase, the mother and daughter centrioles are held together by the centrosome linker (or G1–G2 tether) (Nigg and Stearns, 2011). Following G1, this linker connects the duplicated centrosomes in S and G2 phases to ensure organelle cohesion. At late G2/early mitosis, the linker is dissolved through a process called centrosome disjunction, which allows the duplicated organelles to separate and migrate towards opposite poles for the establishment of the mitotic spindle. The timing of linker dissolution is critical for the duration and fidelity of mitosis (Kaseda et al., 2012; Nam and van Deursen, 2014; Silkworth et al., 2012; Zhang et al., 2012). Accelerated or delayed centrosome disjunction is thought to induce chromosome lagging, resulting in aneuploidy and cancer. It can also cause spindle misorientation, leading to alteration of balance between symmetrical and asymmetrical division and hence, cell renewal and differentiation (Nam et al., 2015).

The centrosome linker is a proteinaceous structure whose molecular composition, architecture and assembly mechanism are not fully understood. A handful of proteins known to be involved in linker biology include C-Nap1 (Cep250) (Fry et al., 1998a; Mayor et al., 2000), rootletin (CROCC) (Bahe et al., 2005; Yang et al., 2006), LRRC45 (He et al., 2013), Cep68 (Graser et al., 2007), CCDC102B (Xia et al., 2018), centlein (Fang et al., 2014), Cep215 (Cdk5rap2) (Barrera et al., 2010; Graser et al., 2007) and pericentrin (Jurczyk et al., 2004). C-Nap1 localizes to the proximal end of mother and daughter centrioles, where it docks LRRC45 and rootletin. LRRC45 can self assemble into filaments that link the proximal end of mother and daughter centrioles together (He et al., 2013). Likewise, rootletin is an integral component of the linker capable of self assembling into thin filaments (Vlijm et al., 2018). Cep68 bundles rootletin thin filaments into thick filaments and is periodically present along the filaments (Vlijm et al., 2018). It is suspected that CCDC10B may serve a similar role to Cep68. Furthermore, centlein is proposed to act as a molecular bridge between C-Nap1 and Cep68. Finally, Cep215 and pericentrin are PCM components that form toroids around mother centrioles and contribute to centrosome cohesion through poorly defined mechanisms.

Centrosome disjunction is mainly promoted by the protein kinase Nek2A (Fry et al., 1998b). At late G2/early mitosis, Nek2A is phosphorylated and activated by Mst2 (also known as STK3), which in turn is phosphorylated and activated by Plk1 (Mardin et al., 2010). Upon activation, Nek2A phosphorylates several proteins including C-Nap1 (Fry et al., 1998a), rootletin (Bahe et al., 2005), LRRC45 (He et al., 2013), Cep68 (Fang et al., 2014), CCDC102B (Xia et al., 2018) and centlein (Fang et al., 2014), leading to their delocalization from the centrosome and subsequently linker dissolution. In addition to delocalization, Cep68 is also known to undergo ubiquitylation and proteasomal degradation in mitosis (Pagan et al., 2015). However, for most other linker proteins, it is unclear whether and how their protein abundances are regulated.

Here, we found that Cep44 is a novel linker component that maintains centrosome cohesion by specifically controlling the stability of rootletin.

## RESULTS AND DISCUSSION

### Cep44 is a novel protein localized to the proximal end of centrioles

Recent proteomic analyses of human centrosomes have led to the identification of a large number of proteins, some of which remain uncharacterized (Andersen et al., 2003; Jakobsen et al., 2011). Among these, Cep44 is highly conserved among eukaryotes. Full-length human protein contains 390 amino acids (aa) and is predicted to have a coiled-coil domain (aa 235–268) and a low complexity region (aa 335–345). To characterize Cep44, we obtained a polyclonal antibody against Cep44 from a commercial source. Western blot studies revealed that this antibody recognizes a single band of ∼44 kDa in U2OS cell extracts (Fig. S1A). This band was markedly reduced upon depletion of Cep44 with two individual siRNA oligonucleotides, thus confirming antibody specificity (Fig. S1A).

**Fig. 1.**
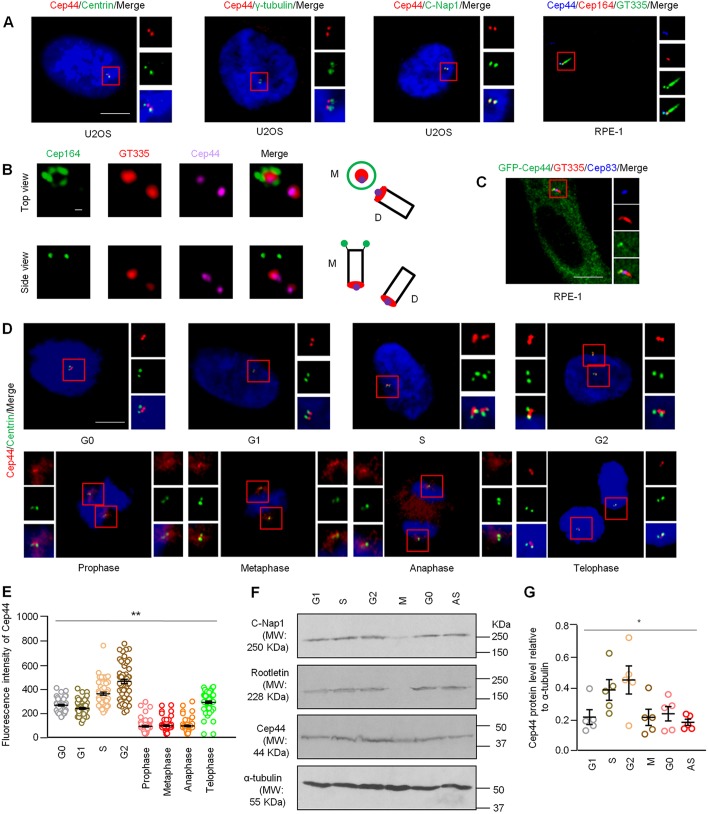
**Cep44 is a proximal end centriolar protein whose level changes during the cell cycle.** (A) U2OS cells or RPE-1 G0 cells were stained with the indicated antibodies with or without DAPI. GT335, glutamylated tubulin. Scale bar: 10 μm. (B) U2OS cells were stained with the indicated antibodies and images were acquired with 3D-SIM. M, mother centriole; D, daughter centriole. Scale bar: 200 µm. (C) RPE-1 G0 cells expressing GFP–Cep44 were stained with the indicated antibodies. Scale bar: 10 μm. (D) RPE-1 cells were stained with DAPI and the indicated antibodies. Scale bar: 10 μm. (E) Quantification of Cep44 fluorescence intensity at the centrosome. Results are mean±s.e.m. based on three independent experiments with 20 cells per experiment (*n*=60). **P<0.01 (one-way ANOVA). (F) RPE-1 cell lysates were western blotted with antibodies against Cep44, C-Nap1 or rootletin. α-tubulin was used as loading control. AS, asynchronous. A representative experiment is shown. (G) Quantification of Cep44 level relative to α-tubulin. Results are mean±s.e.m. based on five independent experiments (*n*=5). *P<0.05 (one-way ANOVA).

To examine the localization of Cep44, we conducted immunofluorescence experiments with antibodies against Cep44 in U2OS or RPE-1 cells and detected a signal that colocalized with the proximal centriolar markers C-Nap1 and glutamylated tubulin (Fig. 1A). This signal did not colocalize with the distal centriolar marker centrin, the mother centriole-specific distal appendage marker Cep164 or the PCM marker γ-tubulin (Fig. 1A). Moreover, the signal was notably absent upon depletion of Cep44 (Fig. S1B), further validating antibody specificity. 3D-SIM studies showed a dot-like pattern of Cep44. In the top view, one Cep44 dot could be found inside the cylinder of Cep164 and overlapped with one glutamylated tubulin dot, while the other Cep44 dot was close to another glutamylated tubulin dot (Fig. 1B). In the side view, Cep44 was located in close proximity to glutamylated tubulin but far away from Cep164 (Fig. 1B). Unlike rootletin or LRRC45, Cep44 did not exhibit a fiber-like appearance (Fig. 1A,B). In addition to endogenous Cep44, we found that recombinant Cep44 expressed in RPE-1 cells also colocalized with glutamylated tubulin (Fig. 1C). These results together suggest that Cep44 localizes to the proximal end of mother and daughter centrioles.

Next, we studied the localization and protein level of Cep44 across the cell cycle in RPE-1 cells. Cep44 antibodies stained two dots in G0 and G1, and four dots in S and G2 phase cells (Fig. 1D), suggesting that the protein localizes to procentrioles in addition to mother and daughter centrioles. During mitosis and, in particular, prophase, metaphase and anaphase, Cep44 dots were less intense and staining became more diffuse (Fig. 1D). Quantification of centrosomal Cep44 intensity showed a moderate signal in G0 and G1, a bright signal in S and G2, and a weak signal in prophase, metaphase and anaphase (Fig. 1E). Likewise, the protein level of Cep44 peaked in S and G2, and dropped in M phase (Fig. 1F,G). Given that the protein levels of C-Nap1 and rootletin were also reduced in mitosis (Fig. 1F), as reported previously for C-Nap1 (Mardin et al., 2010; Mayor et al., 2002), the decrease in Cep44 abundance might be related to the dissolution of the centrosome linker.

### Cep44 is required for centrosome cohesion

**Fig. 2.**
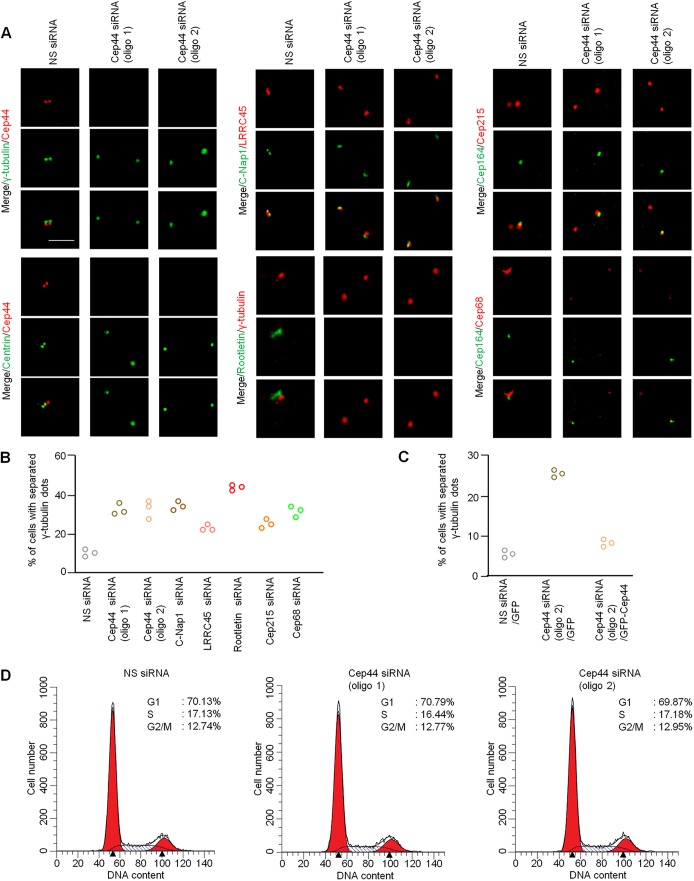
**Cep44 is required for centrosome cohesion.** (A) U2OS cells transfected with non-specific (NS) or Cep44 siRNAs (oligo 1 or 2) were stained with the indicated antibodies. Scale bar: 10 µm. (B) The percentage of U2OS cells with separated γ-tubulin dots is presented. (C) The percentage of GFP-positive U2OS cells with separated γ-tubulin dots is presented. (D) FACS analysis of the cell cycle states of U2OS cells transfected with NS or Cep44 siRNAs. For B,C, three independent experiments were performed, and each experiment involved counting >100 cells for each condition.

**Fig. 3.**
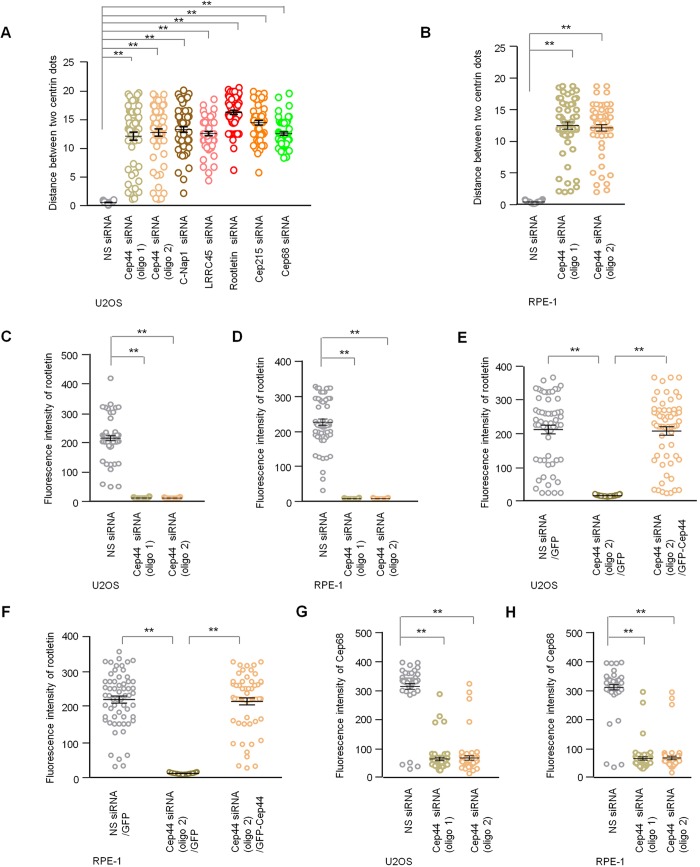
**Cep44 is required for the localization of rootletin to the centrosome.** (A,B) Quantification of the distance between two centrin dots in U2OS or RPE-1 cells. G1 cells with the best knockdown were chosen for quantification. (C,D) Quantification of rootletin fluorescence intensity at the centrosome in U2OS or RPE-1 cells. (E,F) Quantification of rootletin fluorescence intensity at the centrosome in U2OS or RPE-1 cells. GFP-positive cells were chosen for quantification. (G,H) Quantification of Cep68 fluorescence intensity at the centrosome in U2OS or RPE-1 cells. For A–H, results are mean+s.e.m. based on three independent experiments with 20 cells per experiment (*n*=60). **P<0.01 (two-tailed Student's *t*-test).

To explore whether Cep44 plays a role in centrosome cohesion, we depleted Cep44 by using two individual siRNAs. We found that the percentage of Cep44-depleted U2OS cells with premature separation of γ-tubulin dots is much higher than that of control cells (Fig. 2A,B). This phenotype could be rescued by expression of GFP–Cep44 (Fig. 2C), and did not arise from perturbation of the cell cycle (Fig. 2D). Premature separation of γ-tubulin dots is indicative of loss of centrosome cohesion since we observed this in cells depleted of C-Nap1, LRRC45, rootletin, Cep215 or Cep68 (Fig. 2B). Besides γ-tubulin dots, centrin, C-Nap1, LRRC45, Cep215 and Cep68 dots were also found to be prematurely separated (Fig. 2A). Centrosome cohesion was deemed to be lost when the distance between the mother and daughter centriole, which were marked by centrin, exceeded >2 µm (Graser et al., 2007). The mean distance between the two centrin dots was 12.0 µm (oligo 1) or 12.7 µm (oligo 2) in Cep44-depleted cells, compared to 0.6 µm in control cells (Fig. 3A). Moreover, an increased distance between the two centrin dots was found in another cell line, RPE-1, depleted of Cep44 (Fig. 3B). These data indicate that Cep44 plays a role in centrosome cohesion.

### Cep44 controls the stability of rootletin

**Fig. 4.**
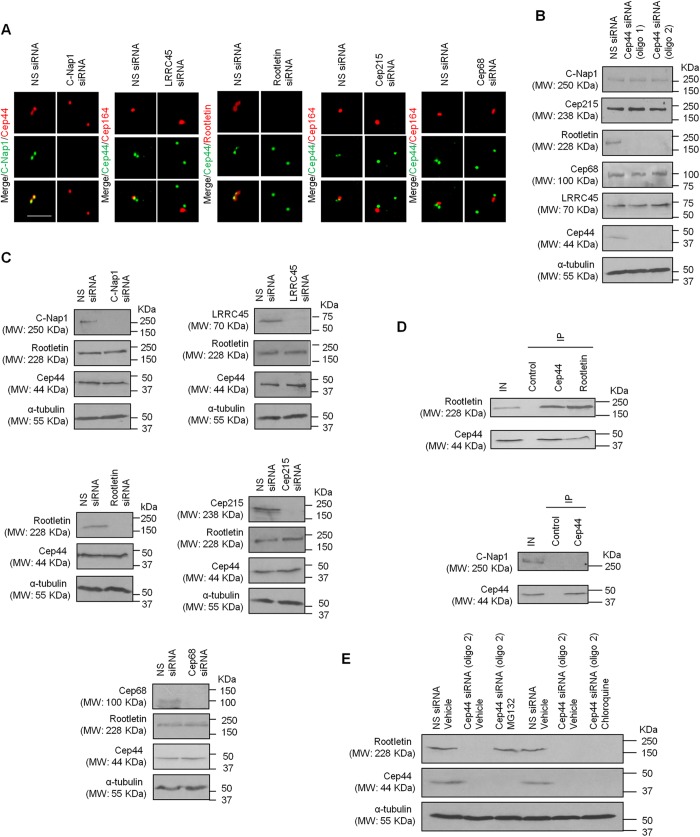
**Cep44 specifically binds to and stabilizes rootletin, and its localization is independent of known linker proteins.** (A) U2OS cells transfected with non-specific (NS), C-Nap1, LRRC45, rootletin, Cep215 or Cep68 siRNAs were stained with the indicated antibodies. Scale bar: 10 µm. (B) U2OS cells were transfected with NS or Cep44 siRNAs (oligo 1 or 2). Lysates were western blotted with the indicated antibodies. α-tubulin was used as loading control. (C) U2OS cells were transfected with NS, C-Nap1, LRRC45, rootletin, Cep215 or Cep68 siRNAs. Lysates were western blotted with the indicated antibodies. α-tubulin was used as loading control. (D) Western blotting of endogenous Cep44, rootletin and C-Nap1 after immunoprecipitation from U2OS lysates with control, anti-Cep44 or anti-rootletin antibodies. IN, input (5 %); IP, immunoprecipitation. (E) U2OS cells were transfected with NS or Cep44 siRNAs and treated with vehicle, MG132 or chloroquine. Lysates were western blotted with the indicated antibodies. α-tubulin was used as loading control.

To address the functional relationship between Cep44 and proteins known to participate in centrosome cohesion, we investigated the effects of depleting Cep44 on the localization of C-Nap1, LRRC45, rootletin, Cep215 and Cep68, and vice versa. Ablation of Cep44 led to a severe loss of centrosomal rootletin (Figs 2A and 3C,D), which could be rescued by GFP–Cep44 expression (Fig. 3E,F), and a significant reduction of Cep68 signal (Figs 2A and 3G,H) without affecting C-Nap1, LRRC45 or Cep215 (Fig. 2A). On the other hand, reciprocal depletion of C-Nap1, LRRC45, rootletin, Cep215 or Cep68 did not impinge on the localization of Cep44 (Fig. 4A). These results indicate that Cep44 and C-Nap1, LRRC45 or Cep215 are independently recruited to the centrosome. Moreover, they suggest that Cep44 is required for proper centrosomal localization of rootletin and to a lesser extent, Cep68.

Next, we investigated the effects of depleting Cep44 on the protein levels of C-Nap1, LRRC45, rootletin, Cep215 or Cep68, and vice versa. The level of rootletin, but not C-Nap1, LRRC45, Cep215 or Cep68, was dramatically reduced upon Cep44 loss (Fig. 4B). In contrast, ablation of C-Nap1, LRRC45, rootletin, Cep215 or Cep68 had no effect on the level of Cep44 (Fig. 4C). Furthermore, depletion of C-Nap1, LRRC45, Cep215 or Cep68 did not alter the level of rootletin (Fig. 4C). Collectively, these data support the notion that Cep44 specifically regulates the stability of rootletin, which in turn impinges on its localization to the centrosome.

Finally, we asked whether Cep44 physically interacts with rootletin and C-Nap1. By conducting immunoprecipitation experiments, we demonstrated co-immunoprecipitation of endogenous Cep44 and rootletin (Fig. 4D). In contrast, Cep44 did not bind to C-Nap1 (Fig. 4D). Thus, it seems likely that Cep44 associates with rootletin to control its stability.

Because the centrosome linker is a highly dynamic structure, we speculate that linker proteins might turn over quickly. One linker component Cep68 has been shown to be ubiquitylated by two ubiquitin ligases, βTrCP and VHL (Pagan et al., 2015; Yin et al., 2017). Although it is known that VHL-mediated ubiquitylation and degradation of Cep68 is inhibited by rootletin and enhanced by MCM7 (Kong et al., 2017; Yin et al., 2017), it remains unclear whether the level of rootletin itself is regulated. Based on our findings, it appears that none of the previously known linker proteins, namely C-Nap1, LRRC45, Cep215 and Cep68, is able to impinge on rootletin protein level. More importantly, we discovered that a novel protein Cep44 is the major player responsible for controlling the abundance of rootletin. Cep44 might stabilize rootletin by preventing rootletin ubiquitylation and/or degradation. Addition of MG132, but not chloroquine, stabilized rootletin upon Cep44 ablation (Fig. 4E), suggesting that rootletin is degraded by the ubiquitin-proteasome system rather than through autophagy.

Since Cep44 controls the stability of rootletin, which influences the stability of Cep68 (Yin et al., 2017), ablation of Cep44 might compromise Cep68 localization and protein level. We indeed found that Cep68 is delocalized from the centrosome upon Cep44 loss; however, the protein level of Cep68 remained unchanged. Thus, the effect of Cep68 provoked by Cep44 depletion is likely indirect and therefore, less severe in comparison with rootletin depletion.

In comparison with Cep44, other linker proteins might employ different mechanisms to control centrosome cohesion. C-Nap1 is known to regulate the localization of rootletin by organizing rootletin into a ring structure with protruding filaments at the proximal end of mother and daughter centrioles (Vlijm et al., 2018). Cep215, on the other hand, might be crucial for rootletin filament formation but dispensable for ring formation (Barrera et al., 2010). In contrast, LRRC45 does not affect the localization of rootletin, and vice versa, and these proteins could presumably form independent filaments between the proximal end of mother and daughter centrioles (He et al., 2013). When cells were individually depleted of Cep44, C-Nap1, LRRC45, rootletin, Cep215 and Cep68, we observed an increase in the percentage of cells with separated γ-tubulin dots (Fig. 2B) and an increase in the distance between the two centrin dots (Fig. 3A), suggesting that these proteins support centrosome cohesion to varying degrees. Nevertheless, in light of a previous report showing that the percentage of cells with split centrosomes upon ablation of rootletin and LRRC45 is comparable to that when rootletin alone is not present (He et al., 2013), there are likely non-redundant and redundant mechanisms regulating centrosome cohesion.

Recently, mutations in C-Nap1, CCDC102B and Cep215 have been shown to be associated with various human disorders including Usher syndrome, Seckel syndrome, high myopia, primary microcephaly and agenesis of the corpus callosum (Bond et al., 2005; Fuster-Garcia et al., 2018; Hosoda et al., 2018; Jouan et al., 2016; Khateb et al., 2014; Kubota et al., 2018; Yigit et al., 2015). It would be interesting to determine whether deficiency in Cep44 or any other linker protein is linked to disease.

## MATERIALS AND METHODS

### Cell culture and plasmids

RPE-1 and U2OS obtained from ATCC (Manassas, VA) were regularly tested for mycoplasma contamination. Cells were grown in DMEM (Wisent Inc., Montreal, QC, Canada, 319-005-CL) and supplemented with 10% FBS (Wisent Inc., 080150) at 37°C in a humidified 5% CO_2_ atmosphere. Human Cep44 cDNA (Dharmacon Inc, Lafayette, CO, MHS6278-202829609) was sub-cloned into mammalian vector pEGFP-C1 to generate pEGFP-C1-Cep44. The construct was verified by DNA sequencing.

### Antibodies

Antibodies used in this study included mouse anti-C-Nap1 [immunofluorescence (IF) 1:250 and western blotting (WB) 1:100; sc-390540, Santa Cruz Biotechnology, Dallas, TX], mouse anti-rootletin (IF 1:1000 and WB 1:100; sc-374056, Santa Cruz Biotechnology), rabbit anti-LRRC45 (IF 1:500 and WB 1:100; HPA024768, Sigma-Aldrich, St Louis, MO), rabbit anti-Cep68 (IF 1:1000 and WB 1:100, 15147-1-AP; Proteintech, Burlington, ON, Canada), rabbit anti-Cep215 (IF 1:1000 and WB 1:500, A300-554A, Bethyl Laboratories, Burlington, ON, Canada), rabbit anti-Cep44 (IF and WB 1:1000; 24457-1-AP, Proteintech), anti-GFP, goat anti-Cep164 (IF 1:500; sc-240226, Santa Cruz Biotechnology), mouse anti-α-tubulin (WB 1:1000; T5168, Sigma-Aldrich), rabbit anti-γ-tubulin (IF: 1:1000, Sigma-Aldrich, T3559), mouse anti-γ-tubulin (IF 1:1000; T6557, Sigma-Aldrich), mouse anti-glutamylated tubulin (GT335) (IF 1:1000; AG-20B-0020, AdipoGen Life Sciences, Burlington, ON, Canada) and mouse anti-centrin (IF 1:1000; 04-1624, Sigma-Aldrich).

### Cell cycle synchronization and fluorescence-activated cell sorting analysis

RPE-1 cells were brought to G0 by serum starvation for 72 h. For synchronization in the G1, S, G2 and M phases, cells were treated with 0.4 mM mimosine for 24 h, with 2 mM hydroxyurea (HU) for 24 h and released for 4 h, with 2 mM HU for 24 h and released for 9 h, and with 40 ng/ml nocodazole for 24 h, respectively. Cell cycle distribution was confirmed by fluorescence-activated cell sorting analysis (Barbelanne et al., 2013). Briefly, cells were fixed in 95% ethanol at 4°C for 1 h and subsequently incubated with 0.1 mg/ml RNase A in PBS at 37°C for 30 min. After centrifugation and removal of supernatant, cells were stained with 50 µg/ml propidium iodide at 4°C for 30 min. Samples of 100,000 cells were analyzed with a FACScalibur flow cytometer (BD Biosciences, San Jose, CA), CellQuest (BD Biosciences) and ModFit (ModFit LT, Topsham, ME) cell cycle analysis software.

### Treatment with proteasome and autophagy inhibitors

Cells were treated with 10 µM MG132 for 6 h or 75 µM chloroquine for 24 h prior to collection.

### Immunoblotting and immunofluorescence

Immunoblotting and immunofluorescence were performed as described previously (Barbelanne et al., 2016). Briefly, cells were lysed in a lysis buffer (50 mM HEPES pH 7.4, 250 mM NaCl, 5 mM EDTA, 0.1% NP-40, 1 mM DTT, 0.1 M AEBSF, 2 μg/ml leupeptin, 2 μg/ml aprotinin, 10 mM NaF, 50 mM β-glycerophosphate and 10% glycerol) at 4°C for 30 min. Extracted proteins were recovered in the supernatant after centrifugation at 16,000 ***g*** for 5 min. For immunoblotting, 100 μg of extract was used and proteins were analyzed by SDS-PAGE and immunoblotted with primary antibodies, followed by horseradish peroxidase-conjugated secondary antibodies (Rockland Inc., Mississauga, ON, Canada, 610-703-002 and 611-7302). For immunofluorescence staining, cells were fixed with cold methanol or 4% paraformaldehyde and permeabilized with 1% Triton X-100 in PBS. Slides were blocked with 3% BSA in 0.1% Triton X-100 in PBS and subsequently incubated with primary antibodies and secondary antibodies. Secondary antibodies used were Cy3- (Jackson Immunolabs, Burlington, ON, Canada, 711-165-151 and 715-165-152), DyLight649- (Jackson Immunolabs, 715-495-151) or Alexa Fluor 488- (Thermo Fisher Scientific, Saint-Laurent, QC, Canada, A11008, A11055 and A11001) conjugated donkey anti-mouse, anti-goat or anti-rabbit IgG. DAPI (Molecular Probes, Saint-Laurent, QC, Canada, D3571) stained DNA and slides were mounted, observed and photographed using a Leitz DMRB (Leica, Concord, ON, Canada) microscope (100×, NA 1.3) equipped with a Retiga EXi cooled camera. Super-resolution 3D-SIM imaging was performed by using an ELYRA PS.1 microscope (Carl Zeiss, Toronto, ON, Canada) equipped with an alpha Plan-Apochromat 100×/1.46 NA oil DIC M27 immersion objective and 488 nm, 561 nm and 640 nm lasers (Hossain et al., 2019). Image stacks of 2 µm in height with a *z*-distance of 0.116 µm were acquired with an Andor iXon 885 EMCCD camera. Each *z*-section was recorded with five grating rotation and five phase changes.

### Quantification of fluorescence intensity and protein band

A region of interest was drawn around a fluorescent spot in the vicinity of the centrosome (Hossain et al., 2017). The area of the region of interest was used to determine the fluorescence intensity by using Volocity6 (PerkinElmer, Woodbridge, ON, Canada). Image conditions were identical in all cases and no areas were saturated as confirmed by the pixel intensity range. Protein bands from western blot films were quantified with ImageJ (NIH, Bethesda, MD). Different film exposure lengths were used to prevent saturation.

### RNA interference

Synthetic siRNA oligonucleotides were purchased from Dharmacon and the sequences were:

NS (non-specific), 5′-AATTCTCCGAACGTGTCACGT-3′; C-Nap1, 5′-GAGCAGAGCTACAGCGAAT-3′; rootletin, 5′-AAGCCAGTCTAGACAAGGA-3′; LRRC45, 5′-CCAACAGAACAAGTCCATT-3′; Cep68, 5′-CGAAGATGATCCATCCCTA-3′; Cep215, 5′-GCAAGGATCTGAATTTGTT-3′; Cep44 oligo 1, 5′-GAGGTGGACTGTGTAGGTTTG-3′; and Cep44 oligo 2 (3′-UTR), 5′-GAGCAATGATTATACTGCTTT-3′. siRNA transfection was performed using siIMPORTER (Millipore, Etobicoke, ON, Canada, 64-101) according to manufacturer's instructions. Cells were harvested 72 h after siRNA transfection.

### Statistical analysis

Data analysis was performed using a one-way ANOVA or two-tailed Student's *t*-test on Prism 8 (GraphPad, San Diego, CA) and are indicated as *P<0.05 and **P<0.01.

## Supplementary Material

Supplementary information
