# Supplementary material for: Cep44 functions in centrosome cohesion by stabilizing rootletin
Source: J Cell Sci. 2020 Feb 21;133(4):jcs239616. doi: 10.1242/jcs.239616 (PMC7044459; doi:10.1242/jcs.239616)
Supplement: Supplementary information [file joces-133-239616-s1.pdf]

Supplementary information

Fig. S1: Validation of the Cep44 antibody

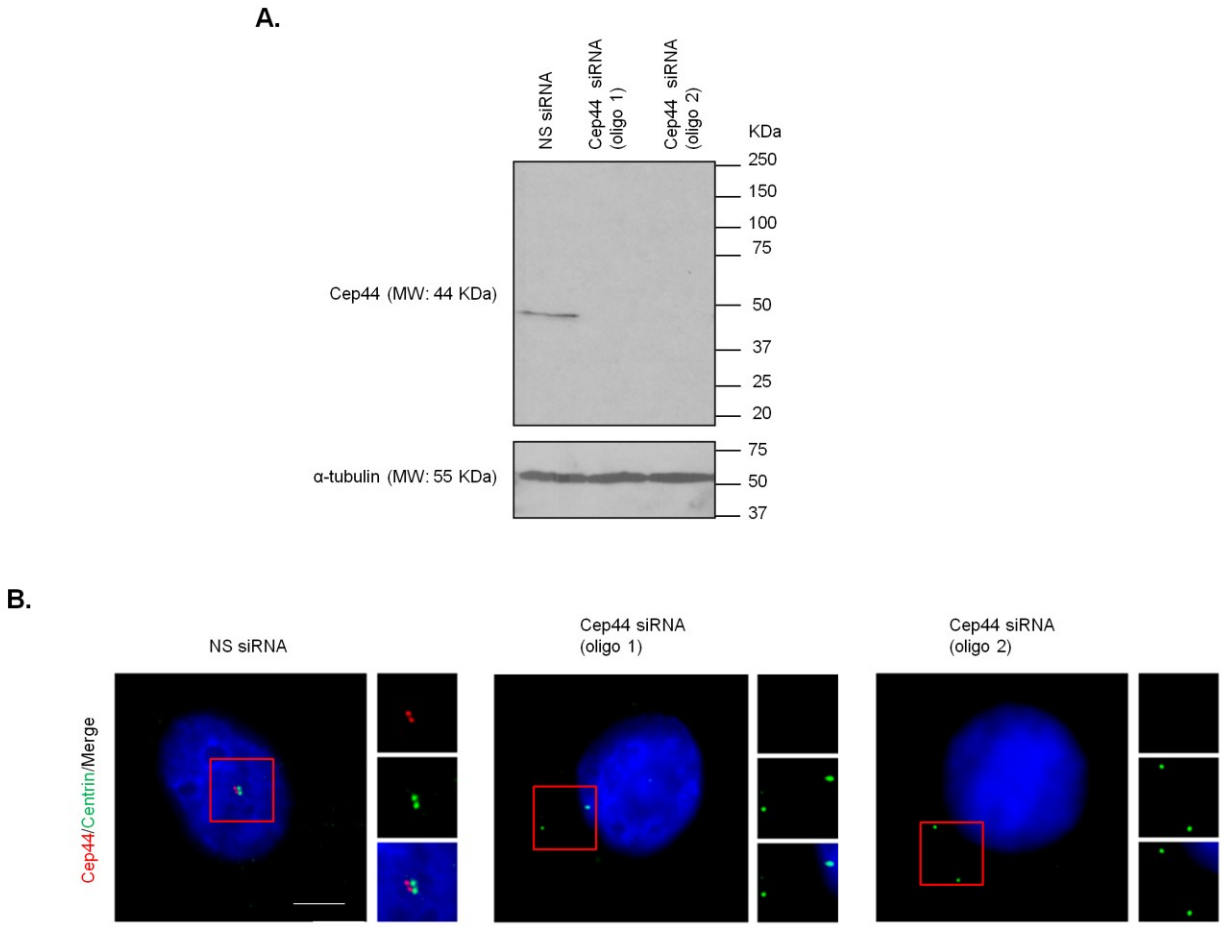

(**A**) U2OS cells were transfected with NS (non-specific) or Cep44 siRNAs (oligo 1 or 2). Lysates were Western blotted with antibody against Cep44. α-tubulin was used as loading control. (**B**) U2OS cells transfected with NS or Cep44 siRNAs were stained with DAPI and the indicated antibodies. Scale bar, 10 μm.
